# Halictine-II as a Potential Broad-Spectrum Antifungal and Antibacterial Agent

**DOI:** 10.3390/ph19091379

**Published:** 2026-09-01

**Authors:** Nick Zhang, Marlys Kutach, Lexis Hsieh, Shelby Vexler, Irene A. Chen

**Affiliations:** 1 Department of Chemical and Biomolecular Engineering, University of California, Los Angeles, CA 90095, USA; 2 Department of Chemistry and Biochemistry, University of California, Los Angeles, CA 90095, USA

**Keywords:** halictine, antifungal, antimicrobial peptide

## Abstract

Antibiotic resistance of microorganisms threatens global health and agriculture. Antimicrobial peptides can display broad-spectrum activity, making them candidate alternatives to antibiotics. Here, ten antimicrobial peptides were tested against fungal pathogens, including *Candida albicans*, *Cryptococcus neoformans*, *Aspergillus niger*, and Gram-negative (*Escherichia coli* and *Pseudomonas aeruginosa*) and Gram-positive (*Staphylococcus aureus*) pathogens. Aurein 1.2, halictine-II, and macropin-I showed broad-spectrum activity. In particular, halictine-II showed high potency combined with low hemolytic activity, with the MIC values across species being 5 to 90-fold lower than the observed hemolytic concentration.

## 1. Introduction

Antimicrobial peptides (AMPs) are a promising class of antibacterial and antifungal agents to address the rise in multidrug-resistant organisms [1]. Although fungal pathogens, particularly *Candida*, *Cryptococcus*, and *Aspergillus*, present a special risk due to the small number of antifungal classes (three), the antifungal properties of AMPs have been relatively understudied compared to their antibacterial properties. Some AMPs exhibit broad-spectrum activity due to a general mechanism of membrane disruption by a cationic, hydrophobic peptide. To characterize antifungal activity, we selected ten peptides with varying net charge (−1 to 9), length (9–25 amino acids), and hydrophobicity (32–62%) [2]. The selected peptides (Table 1) are unmodified, with the exception of amidation at the C-terminus in some cases. Most of these peptides display an α-helical secondary structure in aqueous solution, while others are unstructured random coils that may adopt defined secondary structures in the presence of lipids [3,4,5]. This set of peptides is relatively low in molecular weight and possesses limited modifications, which may be advantageous for future synthetic biology applications, while exploring a range of charge and hydrophobicity. All peptides are reported to have anti-Gram-negative and anti-Gram-positive activity, and all but Cr-ACP1 and Cn-AMP1 are also reported to have antifungal activity, but testing has been limited to a few species (references in Table 1). In addition, reported Minimum Inhibitory Concentrations (MICs) vary substantially, possibly due to variable testing conditions. Here, we directly compare the MICs and Minimum Bactericidal or Fungicidal Concentrations (MBCs or MFCs) of these AMPs against Gram-negative, Gram-positive, and fungal pathogens.

## 2. Results

**Figure 1:**
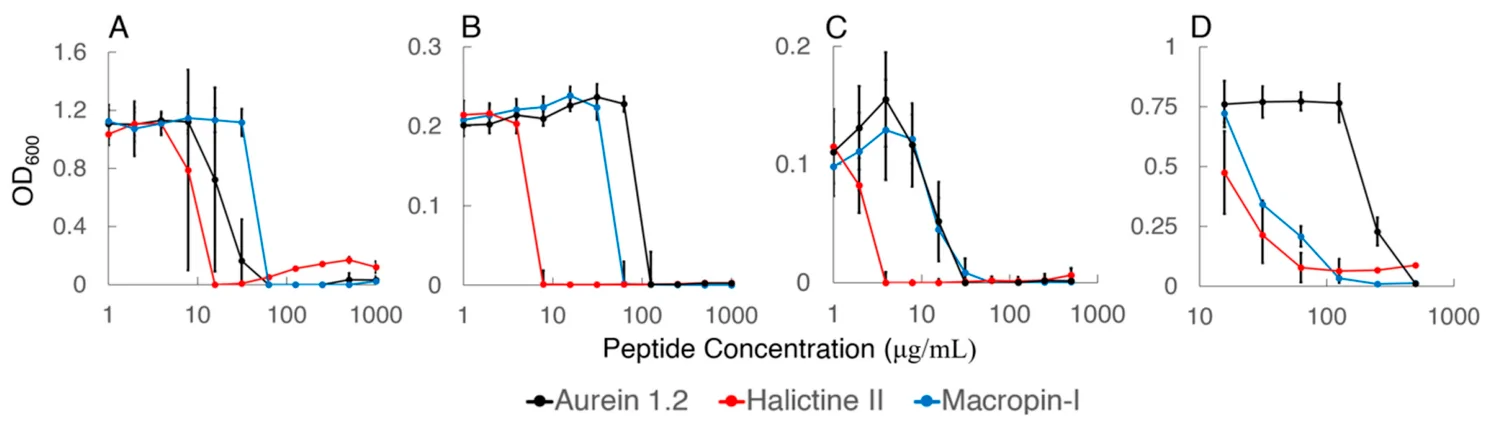
Antifungal activity of AMPs. Endpoint OD_600_ of (**A**) *S. cerevisiae* (*n* = 3), (**B**) *C. albicans* (*n* = 8), (**C**) *C. neoformans* (*n* = 5), and (**D**) *A. niger* (*n* = 4), after incubation with halictine-II, aurein 1.2, or macropin-I at various concentrations. Halictine-II demonstrated poor solubility above 100 μg/mL, contributing to turbidity in some samples. Error bars show standard deviation calculated from replicates.

AMPs were initially assayed against *Saccharomyces cerevisiae* (Table 2). Three AMPs (halictine-II, aurein 1.2, and macropin-I) showed MIC values ≤ 64 μg/mL and MFC values that are two-fold higher than the MIC. Further testing of these AMPs against *C. albicans* and *C. neoformans* gave similar MIC and MFC values (within two-fold) as *S. cerevisiae*. However, activity of these AMPs against *A. niger* was weaker. Growth was also quantified by OD_600_ (Figure 1). The results indicate that halictine-II, aurein 1.2, and macropin-I are inhibitory and fungicidal across different yeasting fungi, and, of these, halictine-II showed a MIC of as low as 4 μg/mL.

To assess the potential of the AMPs as broad-spectrum agents, activities against both Gram-negative (*E. coli*, *P. aeruginosa*) and Gram-positive (*S. aureus*) bacteria were determined. Interestingly, antibacterial activity did not necessarily parallel antifungal activities. For example, jelleine-I showed a low MIC for the Gram-negative bacteria but had poorer activity against *S. cerevisiae*. However, the two most potent antifungal AMPs (halictine-II and aurein 1.2) showed similar activity against bacteria and fungi, and the most potent antifungal AMP, halictine-II, also showed the lowest MIC for bacterial strains (4 to 16 μg/mL).

**Figure 2:**
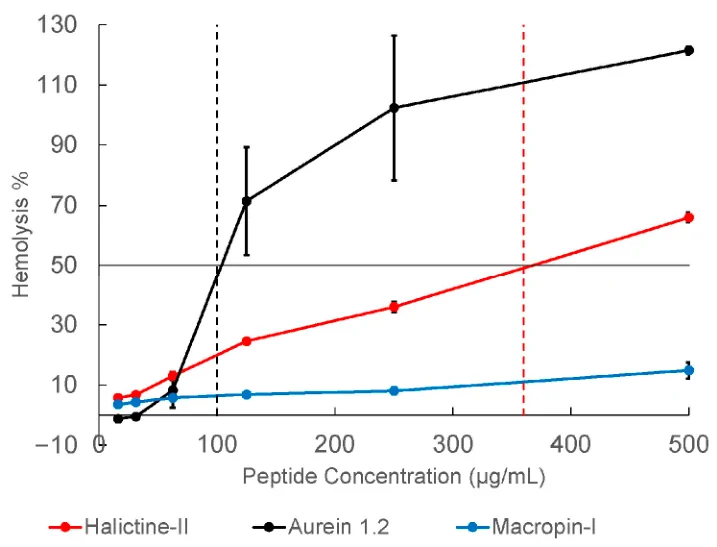
Hemolysis assay of halictine-II, aurein 1.2, and macropin-I with sheep red blood cells at peptide concentrations of up to 500 μg/mL. Error bars show standard deviations calculated from replicates (*n* = 4). Horizontal black line marks 50% hemolysis; dotted vertical lines show interpolated LC_50_ values for aurein 1.2 (black) and halictine-II (red).

The three most effective AMPs were tested for hemolytic properties (Figure 2). Aurein 1.2 displayed the highest hemolytic activity with an estimated LC_50_ of 100 μg/mL, which was within the range of the observed MIC values. Macropin-I showed low hemolytic activity, with less than 15% hemolysis at the highest measured concentration (LC_50_ > 500 μg/mL). Halictine-II showed increasing hemolysis with increasing AMP concentrations, with an estimated LC_50_ of 360 μg/mL, which exceeded the observed MIC values by 5 to 90-fold. Thus, of these three AMPs, halictine-II showed the most promising combination of potent broad-spectrum antimicrobial activity and low hemolytic activity.

## 3. Discussion

Ten AMPs were tested under the same conditions for antimicrobial activity on the model yeast *S. cerevisiae*, as well as Gram-positive and Gram-negative bacterial pathogens, to identify potential broad-spectrum agents. Antifungal and antibacterial activity did not reliably co-occur. Nevertheless, three candidate AMPs (halictine-II, aurein 1.2, and macropin-I) were further tested on fungal pathogen species (*C. albicans*, *C. neoformans*, *A. niger*) and for hemolytic activity. Halictine-II showed a promising combination of broad-spectrum potency and selectivity for pathogen vs. mammalian cells, with MICs of 4–64 µg/mL across all species tested and an LC_50_ for hemolysis (360 µg/mL) that was 5- to 90-fold above the MIC values. At 12 residues, halictine-II is also substantially shorter than other well-studied pore-forming AMPs.

In some cases, MICs differed from the literature values. Cn-AMP1, for example, showed no antimicrobial activity in our assays, which was consistent with one study in which the peptide showed no activity against *E. coli*, *S. aureus*, *P. aeruginosa*, *S. enterica*, *S. epidermidis*, or *Enterococcus faecalis* [5]. However, in a different study, Cn-AMP1 showed promising activity against *E. coli*, *S. aureus*, and *P. aeruginosa*, as well as some fungal species [16]. While the exact cause of discrepancies in antimicrobial activity measurements is unknown, different preparations of the same peptide may vary in activity for multiple reasons, such as synthetic methods affecting counterions, purity and stability. In addition, MIC definitions can vary. In antifungal susceptibility testing, the MIC can be determined within a four-point turbidity scale [18]. While the MIC for amphotericin B is determined at a score of 0, azole MICs are determined at a score of 2 (50% inhibition of growth). Given the slope of OD_600_ vs. peptide concentration observed (Figure 1), different definitions could result in up to four-fold differences in reported MIC values. Similarly, discrepancies between the literature values for hemolytic activity may occur due to procedural differences, as our experiments utilized red blood cells from sheep, while other studies used rat [6], horse [11], or human [6,12] red blood cells.

All three peptides displaying broad-spectrum activity have alpha-helical structures, modest length (12–13 residues), relatively high proportion of hydrophobic residues (50–62%), a range of net charge (+1 to +4.1), and amidation at the C-terminus, which has been demonstrated to be important in increased bioactivity and stability [19]. The antimicrobial mechanism is believed to be initiated by electrostatic interactions followed by insertion and pore formation in the cell membrane [6,7,8,9,10,11,12]. This mechanism is also a concern for hemolysis. Indeed, the antifungal MICs we observed for aurein 1.2 are higher than the previously reported hemolytic concentration (LC_50_ = 44 μg/mL) [11], and our measurement of LC_50_ for aurein 1.2 (100 μg/mL) also fell within the range of MICs we observed. The macropin-I MICs measured here, with the exception of *S. aureus*, are generally lower than the reported hemolytic concentration (LC_50_ = 230 μg/mL) [12], consistent with our observation of low hemolytic activity for macropin-I. However, macropin-I showed consistently lower potency than halictine-II across the fungal and bacterial species tested here. The MICs of halictine-II measured here are all less than the reported hemolytic concentration (LC_50_ = 110–130 μg/mL) [6] as well as our observation of LC_50_ (360 μg/mL).

There are currently four antimicrobial peptides, classified as echinocandins, that are approved for use with fungal infections. These glycosylated or lipo-peptides weaken fungal cell walls by inhibiting beta-1,3-D-glucan synthase, leading to osmotic lysis [20]. In this work, we focused on membrane disruption as a different antimicrobial mechanism due to its broad-spectrum potential. A key advantage of membrane-targeted pore formation as a mechanism is its generalizability across microbial kingdoms, such that potent antifungal activity and antibacterial activity can co-occur. In comparison, antifungal peptides with intracellular targets, such as histatin-5, may engage additional cellular processes, such as energy-dependent internalization [21,22], and their activity may therefore be less robust to different species or conditions [23]. Other well-studied pore-forming peptides include LL-37 (37 aa) and magainin 2 (23 aa). While potent against both Gram-positive and Gram-negative bacteria (MICs of 1 to 10 µg/mL for several species), the cathelicidin LL-37 has less potent antifungal activity (*C. albicans* MIC: 68 µg/mL) and exhibits eukaryotic cytotoxicity within the same range (significant DNA fragmentation at 27 µg/mL), leaving a narrow therapeutic window [24,25,26]. The AMP magainin-2 is also a promising antibacterial agent (MICs of 5–100 µg/mL across several species) [27], but its lower antifungal potency under physiological conditions presents a therapeutic challenge for such infections. From the present in vitro study, halictine-II compares favorably with these established pore-forming peptides in both broad-spectrum (antibacterial and antifungal) potency as well as selectivity, with hemolytic concentrations comfortably exceeding the observed MICs. The short length of halictine-II (12 aa) may also provide practical advantages in synthesis and biodistribution. Overall, the apparent broad-spectrum potency of halictine-II combined with relatively low hemolytic activity makes it a promising candidate for further exploration.

## 4. Materials and Methods

AMPs (Novopro Biosciences, Shanghai, China) were tested for activity against the fungal strains *Cryptococcus neoformans* 32045 (ATCC, Manassas, VA, USA), *Aspergillus niger* 16888 (ATCC), *Candida albicans* 14053 (ATCC), and *Saccharomyces cerevisiae* YJB80 (Jun Park lab, Los Angeles, CA, USA), as well as the bacterial strains *Escherichia coli* 25922 (ATCC), *Staphylococcus aureus* 29213 (ATCC), *Pseudomonas aeruginosa* 25102 (ATCC). *A. niger*, *C. albicans*, *C. neoformans*, and *S. cerevisiae* were grown in ATCC M3 Nutrient Broth, and *E. coli*, *P. aeruginosa*, and *S. aureus* were grown in Mueller Hinton Broth (MHB) [28,29,30]. For *A. niger*, the liquid culture was vortexed to break up mycelia and then passed through a syringe with a sterile gauze plug to remove large aggregates. AMPs were dissolved in ultra-pure water (5 mg/mL) and diluted to a starting concentration of 500 or 1000 μg/mL in media for 2-fold serial dilution in a 96-well plate. A total of 50 μL of culture (OD_600_ = 0.01) was added into each well for a final volume of 100 μL and incubated at 25 °C for *C. neoformans* or 30 °C for *A. niger*, *C. albicans*, and *S. cerevisiae* for 48–72 h, or at 37 °C for *E. coli*, *P. aeruginosa*, and *S. aureus* for 24 h. The wells were assessed for growth visually and confirmed by OD_600_ for fungal strains and *E. coli* using an Infinite 200 Pro plate reader (Tecan, Männedorf, Switzerland). The lowest AMP concentration at which all three replicates had an OD_600_ ≤ 0.01 relative to the sterility control was determined as the MIC. To determine MBC or MFC, 50 μL from the two lowest concentrations showing no visible growth were plated onto LB or YPD agar and grown following ATCC recommendations for each strain. The lowest AMP concentration resulting in no growth on plates was determined as the MBC or MFC.

For the hemolysis assay, three mL of 10% sheep red blood cells (MP Biomedicals, Santa Ana, CA, USA) was gently mixed with 14 mL of PBS at pH 7.4 and centrifuged for 10 min at 800× *g*, at 4 °C. Pelleted cells were washed four times with 14 mL of fresh PBS and, after the final wash, 200 µL of cells were removed and mixed with 9.8 mL of PBS. Two-fold dilutions starting at 500 µg/mL of each AMP was prepared with PBS in a 96-well round-bottom plate, along with a negative control (PBS) and a positive test control (20% Triton X-100 in PBS) at a starting volume of 100 µL. A total of 100 µL of red blood cell solution was added to each well, and the plate was incubated at 37 °C for one hour. After incubation, the plate was centrifuged at 1700× *g* for 5 min, and then 100 µL of the supernatant was transferred to a 96-well flat-bottom plate. Absorbance at 405 nm was measured, and % hemolysis was calculated as 100% × (A_AMP_ − A_PBS_)/(A_TritonX_ − A_PBS_). Where possible, the LC_50_ value was estimated by linear interpolation between the two data points closest to 50% hemolysis.

## Figures and Tables

**Table 1:** Characteristics of selected AMPs [2]. C-terminal amidation is noted in the sequence when present. % Hyd is the percentage of hydrophobic residues. Net charge is calculated at pH 7.

Name	Sequence	Source	Net Charge	% Hyd	Length	Structure
halictine-II [6,7]	GKWMSLLKHILK-NH_2_	*Halictus sexcinctus* (six-banded furrow bee)	4.1	50	12	helical
aurein 1.2 [8,9,10,11]	GLFDIIKKIAESF-NH_2_	*Litoria aurea* (green and golden bell frog)	1	53	13	helical
macropin-1 [12]	GFGMALKLLKKVL-NH_2_	*Macropis fulvipes* (yellow loosestrife bee)	4	62	13	helical
jelleine-I [3]	PFKLSLHL-NH_2_	*Apis mellifera* (western honey bee)	2.1	50	8	random coil
jelleine-II [3,13]	TPFKLSLHL-NH_2_	*Apis mellifera* (western honey bee)	2.1	44	9	random coil
eCATH-1 [4]	KRFGRLAKSFLRMRILLPRRKILLAS	*Equus ferus caballus* (horse)	9	50	25	random coil
scolopin II [14]	GILKKFMLHRGTKVYKMRTLSKRSH	*Scolopendra mutilans* (Chinese red-headed centipede)	8.2	32	25	helical
VCT-VT2 [15]	FLPIIGKLLSG	*Vespa tropica* (greater banded hornet)	1	54	11	helical
Cn-AMP1 [5,16]	SVAGRAQGM	*Cocos nucifera* (coconuts)	1	44	9	random coil
Cr-ACP1 [17]	AWKLFDDGV	*Cycas revoluta* (sago palm seeds)	−1	55	9	helical

**Table 2:** MIC, MFC, and MBC of selected AMPs. Values are given in μg/mL. Colors correspond to MIC activity (red: high activity; see legend). MFC and MBC values are given in brackets. See also Figures S1 and S2. “Lit.” indicates values from the cited literature. Data from different references is separated by semicolons.

	Fungi								Gram-				Gram+	
Strain	*S. cerevisiae* YJB80		*C. albicans* ATCC 14053		*C. neoformans* ATCC 32045		*A. niger* ATCC 16888		*E. coli* ATCC 25922		*P. aeruginosa* ATCC 25102		*S. aureus* ATCC 29213	
AMP	MIC (MFC)	Lit. MIC	MIC (MFC)	Lit. MIC	MIC (MFC)	Lit. MIC	MIC (MFC)	Lit. MIC	MIC (MBC)	Lit. MIC	MIC (MBC)	Lit. MIC	MIC (MBC)	Lit. MIC
halictine-II [6,7]	16 [32]		8 [16]	10	4 [8]		64 [64]		8 [16]	4	8 [8]	62	16 [32]	12
aurein 1.2 [8,9,10,11]	64 [128]	125	128 [250]	125	32 [64]	>1000	>500	>1000	64 [128]	24; 100; 200	128 [128]	95; >200	64 [128]	12; 25
macropin-1 [12]	64 [128]		64 [128]	36	32 [64]		128 [128]		128 [250]	76	128 [128]	7	500	62
jelleine-I [3]	128 [250]								16 [32]	2.5–32	32 [32]	8–62	128 [128]	8–128
jelleine-II [3,13]	128 [250]								64 [125]	15	64 [64]	15	250 [500]	15
eCATH-1 [4]	>1000								64 [128]	1–16	16 [16]	1–8	64 [64]	1–>32
scolopin II [14]	>1000								250 [1000]	5	128 [128]		>1000	0.5–1
VCT-VT2 [15]	>1000								>1000	20	>1000	40	>1000	5
Cn-AMP1 [5,16]	>1000								>1000	16; >1000	>1000	8; >1000	>1000	16; >1000
Cr-ACP1 [17]	>1000								>1000	41	>1000	41	>1000	

MIC(mg/mL)481632641282505001000

## Data Availability

The raw data supporting the conclusions of this article will be made available by the authors on request.

## Supplementary Materials

The following supporting information can be downloaded at: https://www.mdpi.com/article/10.3390/ph19091379/s1, Figure S1: Antibacterial activity of AMPs on *E. coli*; Figure S2: Antifungal activity of AMPs on *S. cerevisiae*.
