# Supplementary material for: Halictine-II as a Potential Broad-Spectrum Antifungal and Antibacterial Agent
Source: Pharmaceuticals (Basel). 2026 Sep 1;19(9):1379. doi: 10.3390/ph19091379 (PMC13610629; doi:10.3390/ph19091379)
Supplement: Supplementary file 1 [file pharmaceuticals-19-01379-s001.zip › pharmaceuticals-4396451-supplementary.pdf]

# Supplementary Information

## Halictine-II as a potential broad-spectrum antifungal and antibacterial agent

Nick Zhang[1], Marlys Kutach[1], Lexis Hsieh[1], Shelby Vexler[1,2], Irene A. Chen[1,2*]

1. Department of Chemical and Biomolecular Engineering, University of California, Los Angeles, USA 90095

2. Department of Chemistry and Biochemistry, University of California, Los Angeles, USA 90095

* Correspondence: ireneachen@ucla.edu

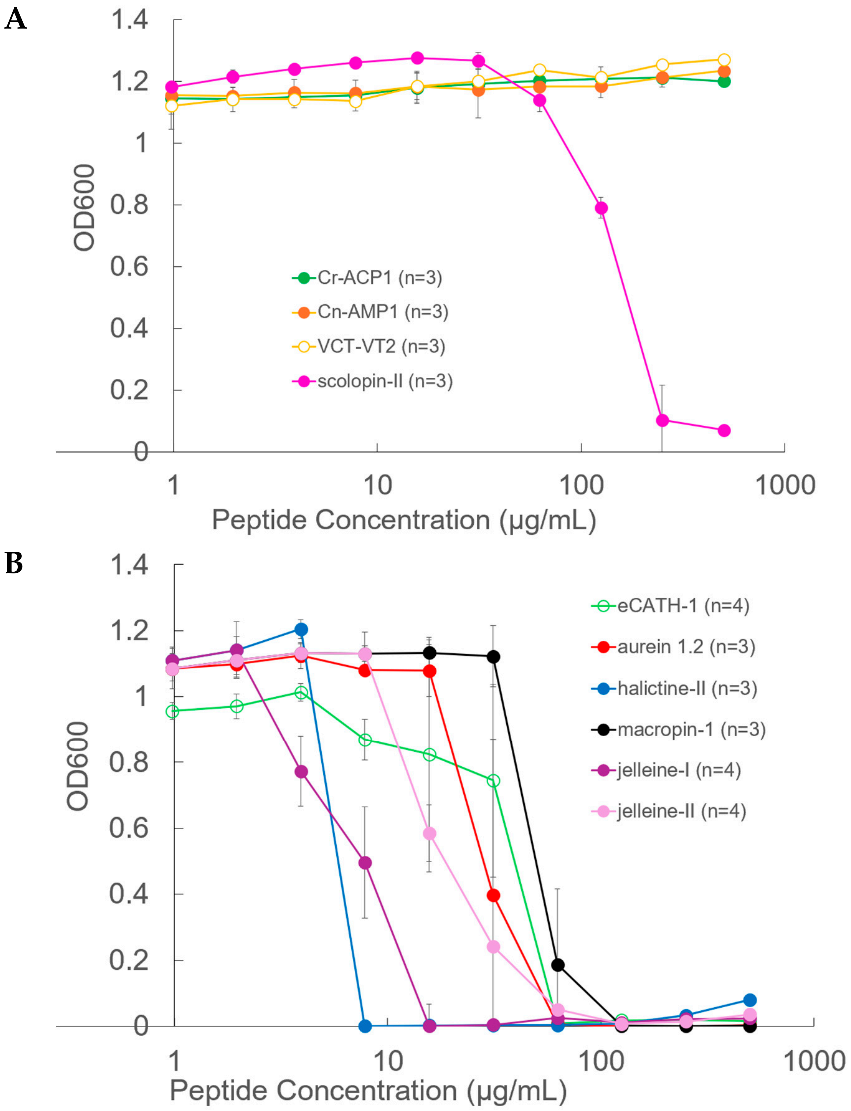


**Figure S1.** Antibacterial activity of AMPs on *E. coli*.

Endpoint $OD_{600}$ of *E. coli* after incubation with **(A)** Cr-ACP1, Cn-AMP1, VCT-VT2, or scolopin-II, and **(B)** eCATH-1, aurein 1.2, halictine-II, macropin-1, jelleine-I, or jelleine-II, at various concentrations. Halictine-II demonstrated poor solubility above 100 μg/mL, contributing to turbidity in some samples. Error bars show standard deviation calculated from replicates.

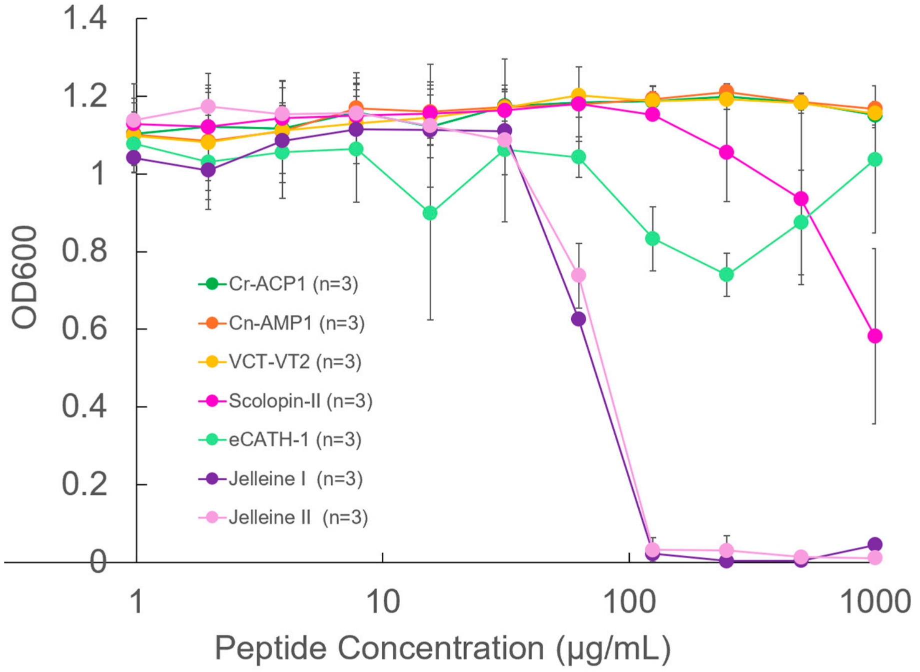


**Figure S2.** Antifungal activity of AMPs on *S. cerevisiae*.

Endpoint $OD_{600}$ of *S. cerevisiae* after incubation with Cr-ACP1, Cn-AMP1, VCT-VT2, scolopin-II, eCATH-1, jelleine-I, or jelleine-II, at various concentrations. Error bars show standard deviation calculated from replicates.
